# Differential toxicity of water- and DMSO-extractable PM2.5 fractions in human alveolar organoids

**DOI:** 10.3389/ftox.2026.1943932

**Published:** 2026-09-25

**Authors:** Shasha Yu, Shangyao Liu, Jiajie Pan, Xinyang Guo, Shuyang Dai, Shaopeng Chen, Jie Ma

**Affiliations:** School of Public Health, Wannan Medical University, Wuhu, China

**Keywords:** DMSO-extractable fraction, human alveolar organoids, PM2.5, toxicology, water-extractable fraction

## Abstract

PM2.5 is a major air pollutant associated with the onset and exacerbation of various diseases. However, due to the lack of suitable experimental models, the damage caused by PM2.5 to the organism and its relationship with diseases remain poorly understood. This study aims to establish a toxicity exposure model for PM2.5 using human alveolar organoids (hALOs) and investigate the differences in alveolar damage caused by water-extractable fraction and DMSO-extractable fraction of PM2.5. The results revealed that PM2.5 induced morphological damage in hALOs in a dose- and time-dependent manner, with the DMSO-extractable fraction eliciting more severe damage than the water-extractable fraction. PM2.5-induced increases in ROS levels and cell death were associated with the observed morphological damage. During the damage process, we also observed a sustained decline in the number of AT2 cells within the organoids, accompanied by a slight increase in the number of AT1 cells, suggesting that AT2-to-AT1 transdifferentiation may be occurring. This study helps advance the application of *in vitro* 3D organoid models in toxicological research and facilitate a deeper integration of PM2.5 toxicology and pathology.

## Graphical Abstract

**Figure:**
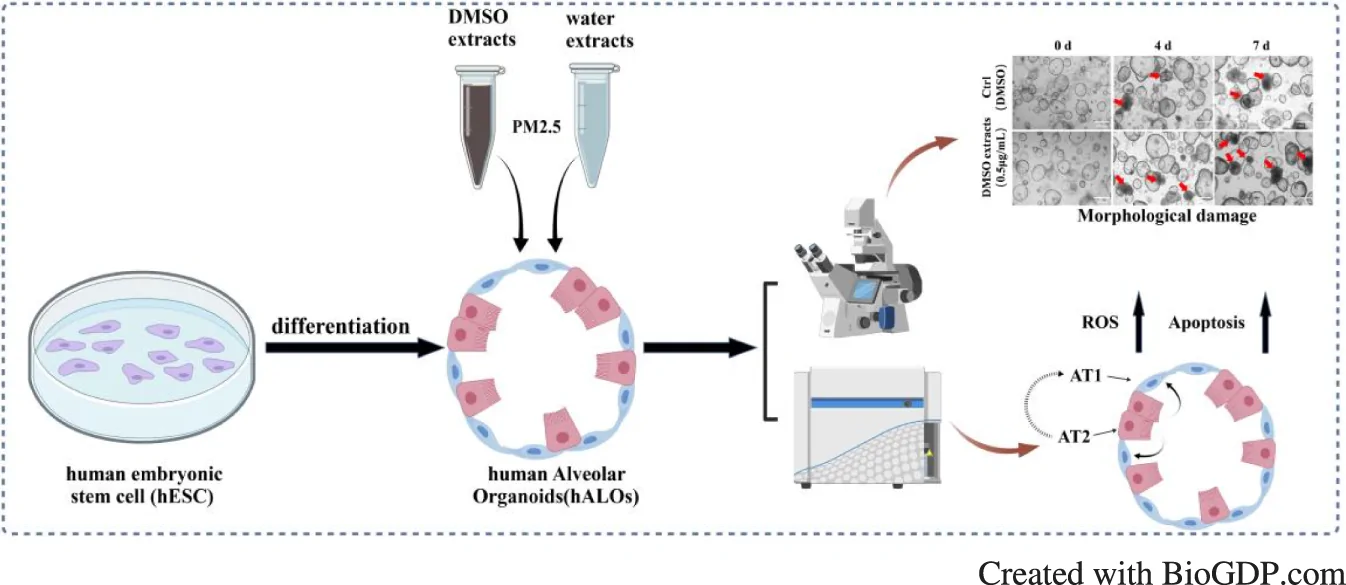

## 1 Introduction

Fine particulate matter (PM2.5, aerodynamic diameter ≤2.5 μm) is a major air pollutant associated with the onset and exacerbation of various diseases. The primary route of exposure to PM2.5 is inhalation. After entering the nasal cavity, PM2.5 travels through the trachea and bronchioles into the alveoli, causing damage to lung tissue and cells, thereby triggering various lung diseases (Sofia et al., 2025)such as asthma, chronic obstructive pulmonary disease (COPD), idiopathic pulmonary fibrosis (IPF), and lung cancer (Cattani-Cavalieri et al., 2025). PM2.5 primarily consists of inorganic, organic, and microbial components (Nasri et al., 2023). The most toxic components of PM are metals, polycyclic aromatic hydrocarbons (PAHs), carbon-containing particles (CPs), and other organic compounds, each of which potentially poses distinct health risks (Wang and Liu, 2023; Li et al., 2025). Previous studies have reported on the health effects of water or DMSO PM2.5 extracts (Zheng et al., 2022; Zhang et al., 2021), however, few studies have examined differences in the health effects between water-extractable fraction and DMSO-extractable fraction of PM2.5.

Current research on lung injury induced or exacerbated by PM2.5 primarily relies on two-dimensional cell line models and rodent models, particularly mice. Due to their lack of structural complexity and dimensionality, two-dimensional cell line models struggle to accurately simulate various cell-cell and cell-matrix interactions. Consequently, they remain insufficient for fully elucidating cellular functions and intercellular signaling dynamics within native tissue microenvironments (Zuniga et al., 2022). Although animal models overcome these limitations, they still face challenges such as species differences from humans, poor reproducibility, and difficulties in real-time monitoring (Zietek et al., 2021). Therefore, new toxicological research models are needed to overcome the limitations of two-dimensional cell models and mouse models and elucidate the pathogenic mechanisms of PM2.5. The emergence of lung organoids has brought new opportunities for in-depth research into toxicological effects.

Organoids are aggregates derived from stem cells or organ progenitor cells, comprising multiple cell types specific to a particular organ. They form through the self-organization of different cell types within a confined space, mimicking *in vivo* growth patterns (Clevers and Hans, 2016). The development of multifunctional human organoid systems has become a focal point in biomedical research, yielding significant benefits for disciplines such as physiology, pathology, pharmacology, and toxicology. Advances in organoid technology are also driving progress in air quality assessment and toxicological research, including studies on PM2.5. A human embryonic stem cell-derived lung progenitor organoid (LPO) model was employed to demonstrate that PM2.5-induced developmental toxicity may be mechanistically linked to dysregulation of the Wnt/β-catenin signaling cascade (Wang et al., 2023); cortical organoids were utilized as a three-dimensional, human-relevant *ex vivo* platform to elucidate the developmental neurotoxicity induced by PM2.5 exposure (Han et al., 2024); and a human retinal organoid model discovered developmental abnormalities elicited by PM2.5 (Zeng et al., 2021). However, toxicological studies based on organoid models are far from sufficient to elucidate multicellular coordination *in vitro*, particularly the multicellular responses following PM2.5 exposure.

Here, we employ a relatively simple alveolar organoid model composed primarily of AT1 and AT2 cells to investigate the alveolar damage caused by PM2.5 exposure and the dynamic changes in AT1 and AT2 cells during this process. We extracted PM2.5 collected in the Wuhu area using both water-extractable fraction and DMSO-extractable fraction, and then applied these two different extracts in *in vitro* hALOs model experiments to investigate differences in their effects on alveolar damage, as well as the time- and dose-response relationships resulting from exposure to these fractions. This study provides new insights into the potential hazards of air pollutants to lung health and offers a powerful tool for developing targeted prevention and control strategies.

## 2 Materials and methods

### 2.1 Human embryonic stem cells (hESCs) culture

The human embryonic stem cell (hESCs) line H9 were obtained from WiCell Research Institute. Matrigel (absin, abs9498) was diluted in pre-cooled DMEM/F12 at an appropriate ratio, added to 6-well plates (1.5 mL per well) for plate coating. The hESCs were seeded in Matrigel coated 6-well plates with mTeSR™1 medium and fresh medium changed daily. The cultures were maintained in a humidified incubator at 37 °C with 5% CO_2_.

### 2.2 Induction and differentiation of human alveolar organoids (hALOs)

1–7 days: When the confluence of hESCs reached 80%–90%, the medium was changed to 1640 medium containing Activin A (100 ng/mL) and CHIR99021 (2 μM) for 3 days to induce the definitive endoderm (DE) stage. Then, the cells were cultured in Advanced DMEM/F12 medium supplemented with Noggin (200 ng/mL), FGF4 (500 ng/mL), CHIR99021 (2 μM), and SB431542 (10 μM) for 4 days to induce the anterior foregut endoderm (AFE) stage. Fresh medium was replaced daily at the DE and AFE stages.

8–21 days: On day 8, the cells were digested and mixed with Matrigel, and then induced toward the ventral anterior foregut endoderm (VAFE) stage. Cells were maintained in DMEM/F12 medium containing BMP-4 (20 ng/mL), ATRA (0.5 μM), CHIR99021 (3.5 μM), GlutaMAX and 2% B27 for 7 days with medium changes every 3 days. Subsequently, cells were cultured in bud tip progenitor organoids (LPOs) medium: DMEM/F12 supplemented with GlutaMAX, 2% B27, CHIR99021 (3 μM), FGF10 (10 ng/mL), KGF (10 ng/mL) and DAPT (20 μM) for 7 days and were passaged every 3–5 days.

22–35 days: On day 22, the culture medium was replaced with human alveolar organoids (hALOs) induction medium: DMEM/F12 medium supplemented with dexamethasone (50 nM), 8-Br-cAMP (100 μM), IBMX (100 μM), KGF (10 ng/mL), 2% B27, 0.25% BSA, 0.1% ITS, CHIR99021 (3 μM) and SB431542 (10 μM), and cultured continuously for an additional 14 days. Fresh medium was replaced every 3 days, and organoids were passaged every 3–5 days. The culture medium and growth factors are listed in Supplementary Table S1.

### 2.3 PM2.5 collection and extraction

The sampling stations were set up on the third-floor terrace of the Yifu Science and Technology Building in the North Zone of the Binjiang Campus of Wannan Medical University in Wuhu City, Anhui Province. The vertical distance from the ground to the sampling stations was approximately 10 m. The flow rate of the medium-volume ambient air particulate matter sampler was 100 L/min, and sampling was conducted continuously for 66 h. The PM2.5 collection membranes used in this study were from February 2023. PM2.5 samples were collected on quartz fiber collection membrane (QFCM, Whatman, United States) and each membrane was divided into two equal sections for extraction of water-extractable fraction and DMSO-extractable fraction, respectively. The detailed extraction procedure was as follows: membranes were cut into small pieces, soaked in ultrapure water or dimethyl sulfoxide (DMSO), sonicated or shaken (40 min), and then filtered through a 10 μm filter. The exposure concentrations for the water-extractable fraction ranged from 0 to 200 μg/mL, while those for the DMSO-extractable fraction ranged from 0 to 12.5 μg/mL. Each condition was performed in at least triplicate wells.

### 2.4 Immunofluorescence staining

For immunofluorescence staining, organoids were incubated with pre-chilled cell recovery solution (1 mL per well) at 4 °C for 1 h to dissolve the Matrigel. Following three washes with pre-chilled PBS, the organoid pellets were resuspended and fixed in 4% paraformaldehyde for 45 min at 4 °C, with gentle agitation every 15 min. Then, organoids were permeabilized in PBS containing 0.5% Tween-20 at 4 °C for 30 min and blocked in PBST with 3% BSA at room temperature (RT) for 1 h. Samples were incubated with primary antibodies (1:1000) overnight at 4 °C, washed three times with PBST, and then incubated with secondary antibodies at RT in the dark for 1.5 h. Nuclei were stained with Hoechst 33342 for 15 min, and images were acquired using an inverted fluorescence microscope.

### 2.5 Flow cytometry

First, the culture medium was removed from the 48-well plate, and the dome surface was washed with pre-cooled PBS. Cell Recovery Solution (350 μL) was added to each well, followed by incubation at 4 °C on a shaker for 1 h to dissolve the Matrigel matrix. The organoid-containing suspension was collected and centrifuged at 1250 rpm for 5 min at 4 °C, the pellet was rinsed once with PBS. Subsequently, 500 μL TrypLE reagent was added for 15 min digestion at 37 °C. Single-cell dissociation was confirmed under a light microscope, and digestion was terminated by adding 5 mL PBS. The mixture was centrifuged at 800 rpm for 5 min at 4 °C, and the supernatant was completely removed. The procedures for cell fixation and antibody labeling were as follows: Samples was fixed with 4% paraformaldehyde at RT for 10 min and washed with PBS. Then, samples were permeabilized with 0.2% Triton X-100 in PBS for 20 min at RT, and blocked with 1% FBS for 1 h. The samples were incubated with primary antibodies at RT for 1 h and washed three times with PBS. Subsequently, secondary antibodies were incubated at RT in the dark for 1 h, washed with PBS and analyzed by flow cytometry. The primary antibodies are listed in Supplementary Table S2.

### 2.6 Quantitative real-time PCR (qRT-PCR) analysis

Total RNA was extracted using Trizol reagent (Beyotime; Cat. No. R0016), then the isolated RNA was subsequently reverse-transcribed into complementary DNA (cDNA) using the Revert Aid RT Reverse Transcription Kit (Thermo Scientific; Cat. No. K1691). Sample preparation was performed in accordance with the manufacturer’s instructions for Taq SYBR® Green qPCR Premix (Universal) (iScience; Cat. No.: EG20117M), and the qRT-PCR reaction procedure was set as follows: 95 °C for 5 min and 40 cycles of 95 °C for 10 s and 60 °C for 30 s. The relative mRNA expression level of the target gene was calculated according to the 2^−ΔΔCt^ method, GAPDH was used as the control for normalization. The target gene primer sequences used in the experiment are listed in Supplementary Table S3.

### 2.7 Apoptosis assay

After PM2.5 exposure for 3 days, the organoids apoptosis was detected using an apoptosis detection kit (MCE, Cat. No. HY-K1073) following the manufacturer’s instruction. Briefly, 1 mL ice-cold Cell Recovery Solution was added to the organoids, incubated with shaking at 4 °C for 1 h. The mixture was then centrifuged at 1250 rpm for 5 min and the supernatant was discarded. The organoid pellet was washed twice with PBS, 0.25% EDTA-free trypsin was added and incubated at 37 °C for 25 min to dissociate organoids into single-cell suspensions. The Annexin V-Fluor 488 and PI mixture was incubated at room temperature in the dark for 15–20 min and analyzed by flow cytometry.

### 2.8 Intracellular oxidative activity detection

After PM2.5 exposure for 3 days, intracellular oxidative activity levels in hALOs were measured using a Reactive Oxygen Species Detection Kit (Beyotime, Cat. No. S0033S) according to the manufacturer’s instructions. Briefly, remove the culture medium from the organoids, rinse with 1 mL of PBS, add diluted DCFH-DA (final concentration of 10 μM), incubate in a 37 °C incubator for 20–30 min, rinse three times with PBS, and finally observe under an inverted fluorescence microscope.

### 2.9 Statistical analysis

All experiments were performed using organoids derived from at least three independent differentiations (n ≥ 3), and each experiment was conducted in at least three replicate wells (technical replicates, n ≥ 3). Data were processed using SPSS and GraphPad Prism 9.0 software, and presented as the mean ± standard deviation (SD). Statistically significant differences were analyzed by One-Way ANOVA, with significance levels set at P < 0.05 (*), P < 0.01 (**).

## 3 Results

### 3.1 Construction and identification of human alveolar organoids (hALOs)

To investigate the toxic effects of PM2.5 on alveoli *in vitro*, we first established alveolar organoids with Human Embryonic Stem Cells line H9 (hESCs-H9) according to a stepwise differentiation protocol (Chiu et al., 2023) (Figure 1A). Prior to the differentiation induction experiments, the pluripotency of the hESCs-H9 was verified using immunofluorescence and flow cytometry (Supplementary Figure S1). The stages of differentiation were monitored using morphological and immunofluorescence imaging techniques, and the transcription levels of marker genes were assessed by qRT-PCR (Figures 1B–D). After approximately 35 days of induction, the hALOs were successfully constructed.

**FIGURE 1:**
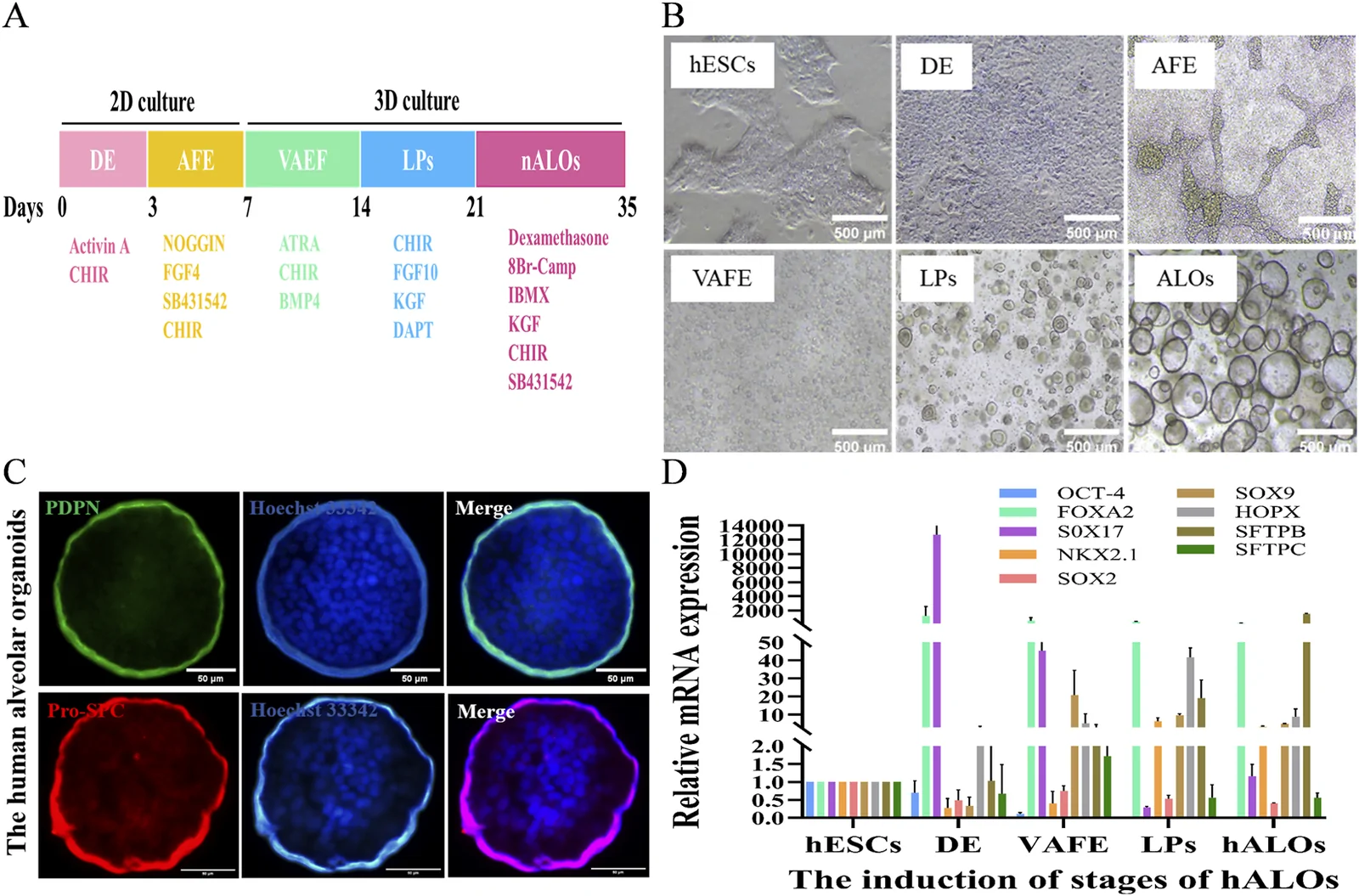
Construction and identification of Alveolar Organoids. **(A)** Schematic flowchart of induced differentiation. **(B)** Bright-field microscopic images showing morphological features at different differentiation stages. **(C)** Immunofluorescence characterization of mature hALOs. PDPN (green) serves as the marker protein for AT1 cells, Pro-SPC (red) marks AT2 cells, and nuclei are stained with Hoechst (blue). **(D)** Relative expression of signature genes at various stages during model induction.

### 3.2 Morphological damage induced by PM2.5 water-extractable fraction and DMSO-extractable fraction

In PM2.5 toxicological studies, organic and inorganic solvents are conventionally employed to extracts PM2.5 from collection membranes. Different solvents frequently alter the compositional profile of the resultant extracts, thereby modulating their ultimate toxicological effects (She et al., 2026). Accordingly, we extracted PM2.5 using pure water and dimethyl sulfoxide (DMSO), respectively, and investigated their toxic effects separately. To characterize chemical entities delivered to organoids, we analyzed elemental composition and PAH content of the PM2.5 extracts. Detailed data are summarized in Supplementary Table S4.

The effects of PM2.5 on morphology were observed by microscope. As depicted in Figure 2A, hALOs prior to PM2.5 exposure exhibited a healthy morphology, characterized by thin walls, smooth edges, translucent central lumens, and relatively uniform sizes. After four days of exposure to the water-extracted PM2.5 fractions, the morphology of the hALOs did not show significant changes, with most remaining healthy, displaying smooth edges and translucent central lumens. After seven days of exposure to the water extracts of PM2.5, the hALOs began to show signs of damage, including dull color, wrinkled edges, and solid central lumens (Figure 2A). Organoids were considered damaged if they displayed shriveled edges and a darkened, solidified central lumen, which could be distinguished from normal organoids. Statistical analysis of the proportion of morphologically damaged hALOs revealed that the number of damaged hALOs increased in a concentration-dependent manner as the exposure concentration rose (Figure 2B). In contrast, the proportion of damaged hALOs in the solvent control group did not change significantly. After 12 days of exposure to the water extracts of PM2.5, the hALOs exhibited severe shrinkage and extensive adhesion among organoids, making it impossible to count the exact number of damaged structures.

**FIGURE 2:**
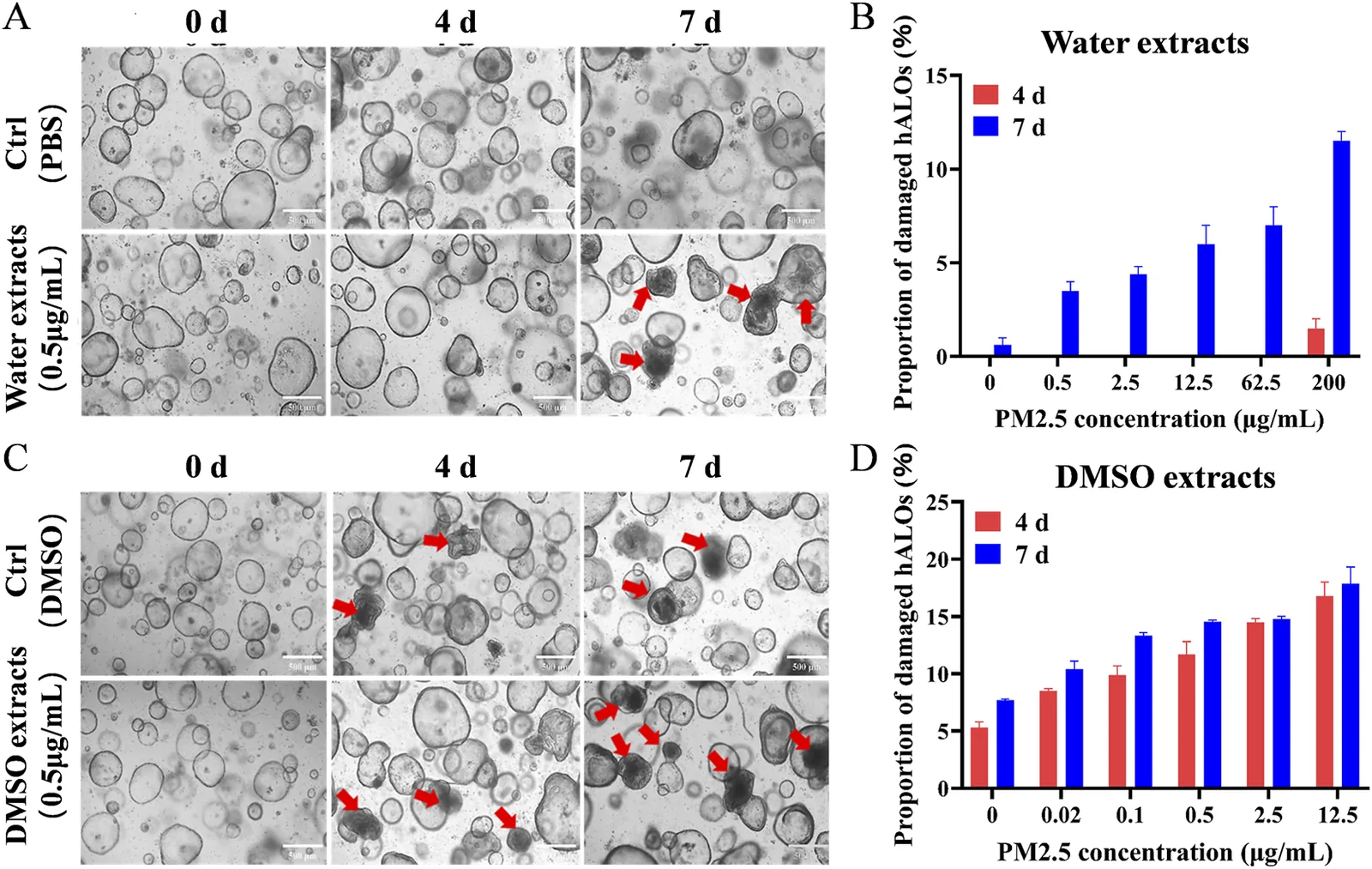
Morphological damage in hALOs. **(A)** Bright-field morphological images of hALOs in water extracts and control group, with red arrows indicating morphologically damaged hALOs. **(B)** Quantitative analysis of the damage proportion of hALOs exposed to water extracts. **(C)** Bright-field morphological images of hALOs in DMSO extracts and control group, with red arrows indicating morphologically damaged hALOs. **(D)** Quantitative analysis of hALOs damage proportion in DMSO extracts.

By day 4, hALOs exposed to DMSO extracts of PM2.5 exhibited a concentration-dependent morphological changes, including dull color, wrinkled edges, and solid central lumens. After sevendays of exposure to the DMSO extracts of PM2.5, the proportion of morphologically damaged hALOs continued to increase with higher exposure concentrations, and the overall proportion was higher than that observed after four days of exposure. After 12 days of exposure to the DMSO extracts of PM2.5, the hALOs showed even more severe shrinkage and large areas of adhesion, making it impossible to count the exact number of damaged structures (Figures 2C,D). Concurrently, an additional hALOs model derived from adult human stem cells (type II alveolar epithelial cells) was also exposed to the DMSO extracts. Following exposure, the morphology of these organoids gradually transitioned from a healthy state characterized by compactness and smooth edges to a damaged state with dull color and indistinct boundaries (Supplementary Figure S2).

A comparison of the data in Figures 2B,D showed that, at the same PM2.5 concentration (e.g., 0.5, 2.5 and 12.5 μg/mL), the morphological changes induced by DMSO extracts were more pronounced than those induced by water extracts, particularly in the 4-day groups. Notably, solvent-control treatment with DMSO as a solvent also caused morphological changes in 5.3% of hALOs, even though we had limited the DMSO concentration to 0.2%. Nevertheless, these results indicate that the proportion of damaged organoids was higher in the groups exposed to DMSO extracts than that in the groups exposed to water extracts, suggesting that the DMSO extracts may possess stronger cytotoxicity.

Beyond morphological changes, we quantified organoid diameter to assess the impact on organoid growth. We found that PM2.5 exposure did not significantly affect the average diameter of surviving organoids (Supplementary Figure S3), a finding that differs from that of Cheng et al. (2024). This may be because new, smaller organoids continued to form throughout the culture process, resulting in no significant difference between the treatment group and the control group.

### 3.3 PM2.5-induced cell death driving morphological transformation of hALOs

To investigate the cause of morphological abnormalities in hALOs induced by PM2.5, we stained the organoids with propidium iodide (PI) after treating them with PM2.5 extracts for 7 days. Significant red fluorescence was observed in organoids treated with water-extractable fraction and DMSO-extractable fraction, indicating a loss of membrane integrity and the presence of dead or dying cells within the organoids (Figure 3A).

**FIGURE 3:**
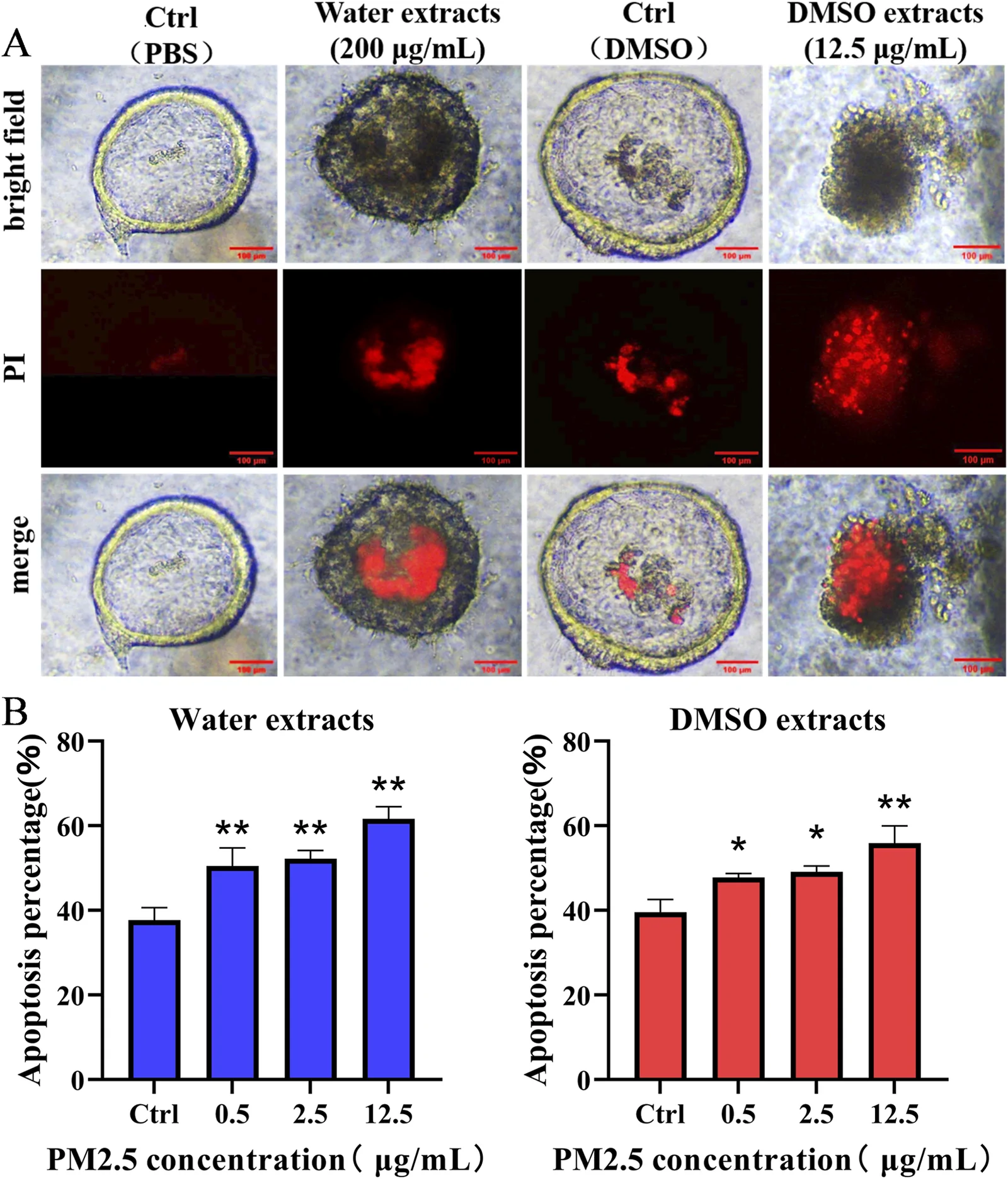
Cell death induced by PM2.5 exposure in hALOs. **(A)** Bright-field images of hALOs exposed to PM2.5 water or DMSO extracts and solvent control, PI staining labels dead cells. **(B)** Apoptosis levels of hALOs exposed to different concentrations of water and DMSO extracts by flow cytometry, stained with Annexin V-Fluor 488 and PI.

Subsequently, the PM2.5-treated hALOs were harvested, trypsinized, labeled with Annexin V/PI and subjected to flow cytometric analysis. Initially, we intended to treat the organoids with a higher exposure concentration for four or 7 days, as in the previous experiments. However, prolonged treatment with high concentrations made the organoids extremely fragile, resulting in significant cell loss during the experiment and rendering them undetectable. Therefore, we conducted this experiment using a lower concentration for 3 days Figure 3B showed both water and DMSO extracts induced dose-dependent apoptosis. At a concentration of 0.5 μg/mL, the proportion of apoptotic cells showed a statistically significant difference.

In terms of apoptosis induction, there was no difference between the water extracts and the DMSO extracts. However, considering the results of the previous *in situ* PI staining experiments on organoids, we speculate that apoptosis is not the primary cause of extensive cell death induced by PM2.5 in the organoids, and other types of cell death may also be involved. Furthermore, during the process of inducing organoid differentiation, cells in the early differentiation stages undergo continuous apoptosis (which explains why the baseline apoptosis rate in organoids is significantly higher than that in two-dimensional cultures, as shown in Supplementary Figure S4), while progenitor cells continue to differentiate and proliferate. We hypothesize that the complex organic compounds contained in PM2.5 may exert a certain inhibitory effect on cell differentiation and proliferation, thereby leading to more severe cell death.

### 3.4 PM2.5 exposure was associated with increased ROS levels

*In vitro* cell culture models have shown that PM2.5 induces an increase in ROS levels (Yuan et al., 2019). However, in three-dimensional organoid models involving multicellular interactions, are ROS also involved in PM2.5-induced damage? To address this, ROS staining was performed on hALOs. Figure 4 showed that exposure to both water extracts and DMSO extracts led to the upregulation of ROS levels in hALOs. The relative fluorescence intensity of hALOs treated with 12.5 μg/mL of DMSO extracts was stronger than that of hALOs treated with 200 μg/mL of water extracts. Because flow-cytometric quantification of DCFH-DA is technically challenging in intact 3D hALOs, we additionally performed assays in the 2D A549 cell line as a supplementary quantitative reference. In A549 cells, DMSO extracts triggered stronger ROS production than water extracts at equal mass concentrations (Supplementary Figure S5). Consistently, DMSO extracts provoked greater ROS induction in hALOs. This phenomenon was consistent with the observed cell death and morphological abnormalities, suggesting a strong association between elevated ROS levels and PM2.5-induced damage to hALOs.

**FIGURE 4:**
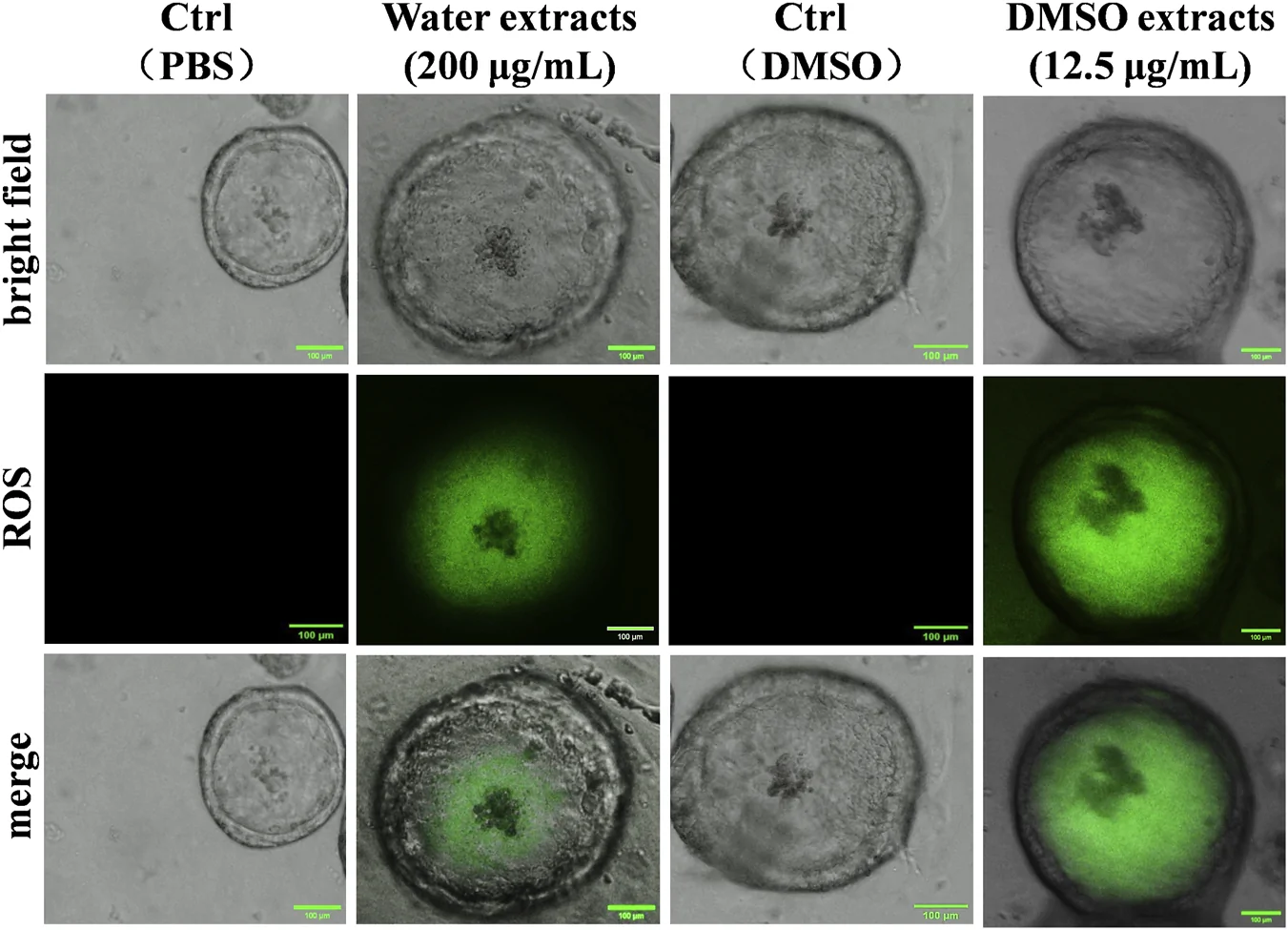
PM2.5-elevated ROS levels in hALOs. The DCFH-DA fluorescent probe was applied to detect intracellular ROS production after water or DMSO extracts of PM2.5 exposure, and all fluorescent images were acquired under fluorescence microscope.

### 3.5 PM2.5-induced alterations in organoid cellular composition

As is well known, alveoli are composed primarily of two distinct cell types: flat AT1 cells, which account for approximately 95% of the alveolar surface and are responsible for mediating gas exchange, and cuboidal AT2 cells, which act as adult stem cells capable of both transdifferentiating into AT1 cells and secreting pulmonary surfactant. The functional integrity of the alveoli depends directly on the extent of damage sustained by these 2 cell types under conditions of external stress.

To further investigate the effects of PM2.5 exposure on the cellular composition of hALOs, we exposed hALOs to water and DMSO extracts for 4′7 and 12 days, respectively, and assessed changes in AT1 and AT2 cells. Immunofluorescence staining was employed to label marker proteins (Podoplanin in AT1 cells and Pro-surfactant protein C in AT2 cells), followed by flow cytometry analysis. Concurrently, qRT-PCR was used to analyze the relative mRNA expression levels of HOPX in AT1 cells and SFTPC in AT2 cells.

Podoplanin (PDPN) serves as a canonical marker of mature AT1 cells and is routinely employed in flow cytometry for the detection of AT1-positive populations (Carignon et al., 2023). exposure to PM2.5 leads to a reduction in PDPN-positive cells. As shown in Figures 5A,C, the proportion of AT1 cells declined in a dose-dependent manner following treatment with either water or DMSO extracts. Notably, the proportion of AT1-positive cells dropped sharply between days 4 and 7, yet rebounded substantially by day 12. In parallel, we assessed transcriptional levels of HOPX, an additional AT1 cell marker gene, by qRT-PCR, and revealed transcriptional changes concordant with the protein-level alterations described above (Figures 5E,G). Although the DMSO extracts exerted a more pronounced effect on AT1 cell proportions and HOPX mRNA levels at day 4 relative to the water extracts, this difference failed to achieve statistical significance.

**FIGURE 5:**
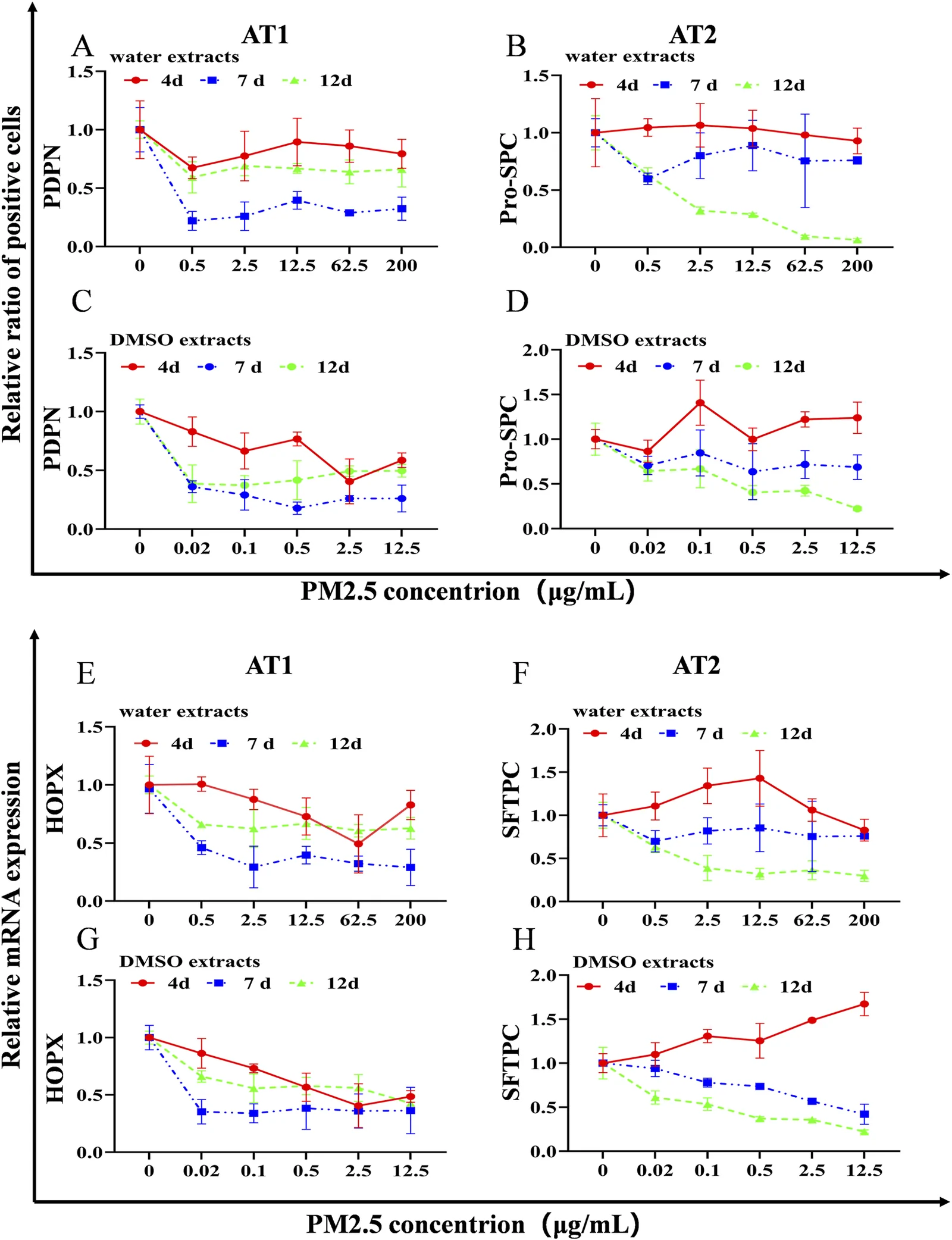
Alterations in the mRNA and protein expression levels of AT1/AT2 marker genes following PM2.5 extracts exposure. **(A–D)** Immunofluorescence staining showing time- and dose-dependent variations in AT1 marker PDPN and AT2 marker pro-SPC under PM2.5 water **(A,B)** and DMSO **(C,D)** fraction exposure. **(E–H)** qRT-PCR quantification of time- and dose-dependent transcription levels of AT1 marker HOPX and AT2 marker SFTPC upon PM2.5 water **(E,F)** and DMSO **(G,H)** fraction exposure.

Pro-surfactant protein C (pro-SPC), encoded by SFTPC, is a canonical AT2 cell marker whose downregulation is implicated in various pulmonary diseases (Beers and Mulugeta, 2017). As shown in Figures 5B,F, 4-day exposure to water extracts elicited no significant alterations in the proportion of pro-SPC-positive cells or SFTPC mRNA levels. Similarly, DMSO extracts exposure for 4 days did not significantly affect the proportion of AT2 cells. However, SFTPC mRNA exhibited a concentration-dependent increase, reaching 1.7-fold of control levels at 12.5 μg/mL (Figures 5F,H). Prolonged exposure resulted in progressive declines in both pro-SPC protein and SFTPC transcript levels. By day 12, AT2 cells treated with 12.5 μg/mL extracts retained only 22% of control levels.

Collectively, both water-extractable fraction and DMSO-extractable fraction of PM2.5 elicited dose-dependent reductions in AT1 and AT2 cell populations, with DMSO extracts exerting more severe effects. While AT2 cell loss was time-dependent, AT1 cells exhibited significant recovery by day 12, suggesting a pattern consistent with the transdifferentiation of AT2 cells into AT1 cells, a process that has been described in other models of lung injury and repair.

## 4 Discussion

Two distinct approaches for alveolar organoid generation—hESCs-based differentiation and adult stem cell-based induction—were employed in this study to establish models optimized for PM2.5 toxicology research (Chiu et al., 2023; Tindle et al., 2021). Although hESCs-derived hALOs are notably time-intensive, hESCs possess high pluripotency and the capacity for unlimited proliferation (Fu and Xu, 2011), supporting long-term culture and dynamic monitoring. Conversely, starting materials derived from adult stem cells are limited, and isolated cells progressively lose their proliferation and differentiation capacity during long-term culture (Kureel et al., 2019). Compared to various existing human lung organoid culture approaches (such as air-liquid interface culture systems (Chen et al., 2024), matrix-based spheroid culture (Umeki et al., 2022), bronchoalveolar-like structures, and lung organoids generated from pluripotent stem cells (Miller et al., 2019)), hESCs-derived hALOs demonstrates higher accuracy in reproducing the composition of human distal alveolar cells. Furthermore, during large-scale *in vitro* culture, these organoids can maintain structural and genetic stability over extended periods. Accordingly, hESCs-derived hALOs were selected as the model system for PM2.5 toxicity assessment in the present study.

PM2.5 is a complex natural mixture comprising diverse organic and inorganic constituents. During extraction from collection membranes, either inorganic solvents (e.g., water, PBS) or organic solvents (e.g., DMSO, ethanol) are typically employed, yielding extracts with distinct compositional profiles. These solvent-dependent variations significantly influence toxicological outcomes (She et al., 2026). Two research groups reported the experimental results of exposing BEAS-2B Cells to PM2.5 inorganic extracts (100–2000 μg/mL) and PM2.5 organic extracts (0–100 μg/mL), respectively (Zhou et al., 2016). One group showed that 250 μg/mL of the inorganic extracts reduced cell viability by 50%; in the other group, about 96 μg/mL of the organic extracts induced the same proportion of cell viability. Although the effective concentration of the latter is substantially lower than that of the former, this disparity alone does not establish superior toxicity of organic extracts over inorganic fractions. Objective assessment of PM2.5 toxicity necessitates simultaneous evaluation of both extracts types within a unified experimental system. In the present study, we selected pure water and DMSO as two representative solvents to extract the water-extractable fraction and DMSO-extractable fraction of PM2.5. As illustrated in Figure 2, DMSO extracts induced more severe morphological abnormalities in hALOs than water extracts under identical exposure concentrations and durations, particularly after 4 days of exposure. Our previous study revealed that the organic components in DMSO-extracted PM2.5 were substantially greater than those in pure water-extracted PM2.5, despite the similarity in inorganic components between the two extracts (She et al., 2026; ZHANG et al., 2016). These differences in organic compounds may be the primary cause of the observed differences in toxicity. The DMSO-extractable fraction, being richer in organic compounds, exerted a more potent inhibitory effect, further highlighting the role of organic components in PM2.5-induced cell toxicity.

However, an unexpected phenomenon was observed in the DMSO solvent exposure group: the DMSO control also induced significant morphological alterations in hALOs. DMSO, a commonly employed organic solvent, is routinely used at concentrations of approximately 10% for cell cryopreservation in two-dimensional culture systems and at 0.01%–1% as a ROS scavenger in toxicological studies to protect cells against ROS-mediated injury. However, the present study revealed that DMSO concentrations as low as 0.2% were sufficient to elicit damage in hALOs, suggesting that the pulmonary toxicity of certain organic solvents may substantially exceed current understanding. While DMSO is a widely used organic solvent in toxicological studies, its cytotoxicity in sensitive 3D models like organoids may be underestimated. This observation raises questions about the suitability of DMSO as an extraction solvent for PM2.5 in organoid-based toxicology. For future studies, less-toxic alternative organic solvents should be considered to minimize confounding solvent-derived interference.

Alveoli represent the smallest functional units of the lung, with gas exchange as their principal physiological role, and are predominantly composed of AT1 and AT2 cells. AT1 cells mediate gas exchange, whereas AT2 cells serve as progenitor cells that replenish AT1 cells via transdifferentiation upon AT1 depletion (Nabhan et al., 2018). Our findings demonstrate that PM2.5 exposure induces alterations in both AT1 and AT2 cell populations. On day 4 of exposure, no significant change in AT2 cell proportion was observed. However, prolonged monitoring revealed that both DMSO and water PM2.5 extracts elicited a marked decline in AT2 cells with increasing exposure duration. In contrast, the proportion of AT1 cells displayed a V-shaped temporal pattern, with the percentage of positive cells increasing by day 12 and surpassing the level observed on day 7 (Figure 5). Studies have shown that air pollutants can impair the regenerative capacity of airway and alveolar stem cells (Wu et al., 2025). Our observation of progressive AT2 cell loss followed by AT1 cell recovery in PM2.5-exposed hALOs aligns with this framework. Therefore, we hypothesize that transdifferentiation from AT2 to AT1 cells occurred by days 7–12. However, we acknowledge that changes in cell population abundance alone cannot establish lineage conversion (Gui et al., 2025). Future studies employing genetic lineage tracing will be necessary to definitively confirm this process in our model. At the same time, no proliferation of AT2 cell was observed in this study. This discrepancy may be attributable to differences in experimental systems and the complex toxicological profile of PM2.5 (Li et al., 2023; Crapo et al., 1980).

Despite its advantages over conventional 2D culture, the hALOs model also has several inherent limitations. The Matrigel-based immersion culture fails reconstruct the physiological air-liquid interface of the lung, potentially modifying the bioavailability of PM2.5 components. Furthermore, PM2.5 toxicity is not restricted to pulmonary tissue; it can trigger systemic adverse outcomes mediated by inflammatory and neuroendocrine pathway cascades (Li et al., 2024). However, the current hALOs model lacks components of immune cells and vasculature, so it cannot reproduce these complex systemic interaction processes. Although there are certain limitations in extrapolating the damage mechanism of the *in vitro* model to humans, the hALOs model remains a powerful tool for bridging the gap between 2D culture and animal experiments.

## 5 Conclusion

In summary, we established a toxicological model utilizing hESCs-derived lung organoids to investigate PM2.5-induced alveolar injury, enabling real-time monitoring and multicellular interaction analysis. Using this model, we demonstrated that DMSO extracts of PM2.5 exhibited markedly greater cytotoxicity and induced more severe morphological alterations in organoids compared to water extracts. Furthermore, both PM_2_._5_ extracts reduced AT1-and AT2-positive cell fractions, consistent with a shifted AT1/AT2 balance that may involve AT2-to-AT1 transdifferentiation.

## Data Availability

The original contributions presented in the study are included in the article/Supplementary Material, further inquiries can be directed to the corresponding authors.
