# Supplementary material for: Differential toxicity of water- and DMSO-extractable PM2.5 fractions in human alveolar organoids
Source: Front Toxicol. 2026 Sep 25;8:1943932. doi: 10.3389/ftox.2026.1943932 (PMC13649214; doi:10.3389/ftox.2026.1943932)
Supplement: Supplementary file 1 [file Supplementaryfile1.docx]

Supporting Information for

**Differential toxicity of water- and DMSO-extractable PM2.5 fractions in human alveolar organoids**

Shasha Yu^a^, Shangyao Liu^a^, Jiajie Pan^a^, Xinyang Guo^a^, Shuyang Dai^a^, Shaopeng Chen^a*^, Jie Ma^a*^

*^a^ School of Public Health, Wannan Medical Universiyt, Wuhu, 241002, P. R. China*

*^*^* Corresponding Authors

Email: [20200068@wnmc.edu.cn](mailto:20200068@wnmc.edu.cn)(Jie Ma); [20200080@wnmc.edu.cn](mailto:20200080@wnmc.edu.cn%20(Shaopeng) (Shaopeng Chen)

**Verification of the pluripotency of hESCs**

The stemness of hESCs refers to their ability to self-renew and their potential for multipotent differentiation (Rizzino and Wuebben 2016). The hALOs models used in this study were derived from hESCs through induced differentiation. Therefore, maintaining the stemness of hESCs is critical for successful organoid construction. To validate the pluripotent identity of hESCs, we detected pluripotency markers OCT-4 and Nanog in hESCs using immunofluorescence and flow cytometry, respectively. The results are shown in **Fig. S1A**, demonstrating high expression of OCT-4 and Nanog in hESCs. Flow cytometry result indicated that a high proportion of double-positive cells compared to the negative control **(Fig. S1B)**, and 92.39% of the cells co-express OCT-4 and Nanog **(Fig. S1C)**. In summary, these results confirmed that the hESCs used in this study maintain intact pluripotent properties.

**Fig. S1.** Verification of hESCs pluripotency. (A) Immunofluorescence staining for pluripotency markers OCT-4 (green) and Nanog (red); nuclei were counterstained with DAPI (blue); (B) Flow cytometric analysis of OCT-4 and Nanog expression. Left: negative control group; right: dual staining of OCT-4 and Nanog; (C) Quantitative statistics of three independent flow cytometry experiments.

**Organoids derived from type II alveolar epithelial cells**

Human alveolar type 2 (AT2) cells were selected to generate human alveolar organoids via a stepwise directed differentiation workflow (**Fig. S2A**). The initial 10 days were used for co-culturing cells with Matrigel to facilitate cell expansion. PneumaCult™ AvOE Seeding Medium was used from day 0 to day 3. Complete PneumaCult™ AvOE Medium was used from days 4-10 with fresh medium change every 3–4 days to obtain mature ATII organoids. Complete PneumaCult™ AvOD Medium from day 10 and fresh medium exchange every 3-4 days. Representative morphological features at different stages are shown in **Fig. S2B**. Human alveolar organoids derived from type II alveolar epithelium were further treated with PM2.5 DMSO extracts. The result showed that the morphology of the organoids gradually changed from a healthy state-characterized by a dense structure and smooth edges-to a damaged state, appearing grayish in color with indistinct boundaries (**Fig. S2C**)

**Fig. S2**. Induction Protocol for Organoids Derived from Type II Alveolar Epithelial Cells and Morphological Damage Caused by PM2.5. (A) Culture workflow of human alveolar organoids derived from type II alveolar cells; (B) Brightfield micrographs of alveolar organoids at different stages; (C) Brightfield micrographs of organoids in control group and DMSO extracts exposure group.

**PM2.5 exposure had no significant effect on organoid size**

For PM2.5-exposed organoids extracted using DMSO, we measured the long axis of individual organoids at 0, 4, and 7 days post-exposure. We found that, because the organoids were continuously generating new, smaller organoids, there was no significant difference in the average diameter of the organoids.

**Fig. S3**. Changes in Organoid Size Before and After PM2.5 Exposure

**Comparison of apoptosis levels in 2D cells and organoids after PM2.5 exposure**

2D cell models and 3D hALO models were treated with water and DMSO extracts of PM2.5 for 3 days. Two cell lines, human bronchial epithelial cells (BEAS-2B) and lung carcinoma cells (A549), were adopted as the 2D cell models. The apoptosis levels of hALOs, BEAS-2B cells, and A549 cells following exposure were detected by flow cytometry. The results showed that after 3 days exposure to PM2.5 water extracts increased the proportion of apoptotic cells in both models in a dose-dependent manner. Even at the lowest exposure concentration of 0.5 μg/mL. significant differences in apoptotic levels were observed in hALOs. By contrast, markedly elevated apoptosis was only detected at higher exposure concentrations in the BEAS-2B cell (62.5 μg/mL) and A549 cell (12.5 μg/mL) models (**Fig. S4A**). After 3 days of exposure to PM2.5 DMSO extracts, apoptosis was observed in hALOs at the low concentration of 0.5 μg/mL, while significant apoptosis occurred in BEAS-2B cells and A549 cells at 12.5 μg/mL and 2.5 μg/mL, respectively (**Fig. S4B**).

**Fig. S4**. Differences in PM2.5-induced apoptosis between hALOs and cultured lung cells. (A) Apoptosis levels of BEAS-2B, A549, and hALOs cells after exposure to PM2.5 water extracts; (B) Apoptosis levels in BEAS-2B, A549, and hALO cells after exposure to PM2.5 DMSO extracts.

**Water- and DMSO-extractable PM2.5 fractions induces ROS production in A549 cells**

A549 cells were exposed to the same concentrations of DMSO and water extracts of PM2.5. Intracellular ROS levels were markedly elevated in A549 cells treated with DMSO-extractable fractions relative to water counterparts, with a statistically significant difference at 12.5 μg/mL **(Fig. S5)**. These data reveal that PM2.5 DMSO extracts exhibit stronger cytotoxic effects compared with water extracts.

**Fig. S5.** Relative intracellular ROS levels in A549 cells exposed to DMSO and water PM2.5 extracts.

**Table S1 The growth factor and culture medium in different stage**

| Stage | Culture medium | Growth factor | Time |
| --- | --- | --- | --- |
| DE | RPMI1640 | Activin A (100 ng/mL)､CHIR99201 (2 μM) | 3 d |
| AFE | Advanced DMEM | Noggin (200 ng/mL)､FGF4 (500 ng/mL)､CHIR99021 (2 μM)､SB431542 (10 μM) | 4 d |
| VAFE | DMEM F12 | BMP4 (20 ng/mL)､ATRA (0.5 μM)､CHIR99021 (3.5 μM)､Glutamax (1%)､B27 (2%) | 7 d |
| LPs | DMEM F12 | Glutamax (1%)､B27 (2%)､CHIR99021 (3 μM)､FGF10 (10 ng/mL)､KGF (10 ng/mL)､DAPT (20 μM) | 7 d |
| ALOs | DMEM F12 | Dexamethasone (50 nM)､8-Br-cAMP (100 μM) ､IBMX (100 μM)､KGF (10 ng/mL)､B27 (2%)､BSA (0.25%)､ITS (0.1%)､CHIR99021 (3 μM)､SB431542 (10 μM) | 14 d |

**Table S2 Primary Antibodies**

| Antibody | Dilution | Manufacturer | Cataloge |
| --- | --- | --- | --- |
| OCT-4 | 1:500 | Santa Cruz Biotech | sc-5279 |
| Nanog | 1:70 | abcam | ab109250 |
| Goat anti-Mouse | 1:50 | ZSGB-BIO | ZF-0312 |
| Goat anti-Rabbit | 1:200 | invitrogen | A-11037 |
| DAPI | 1:50 | Beyotime | C1005 |
| Podoplanin | 1:500 | abcam | ab10288 |
| Prosurfactant Protein C | 1:1000 | abcam | 90716 |
| Goat anti-Mouse | 1:200 | invitrogen | A-21235 |
| Goat anti-Rabbit | 1:200 | yeasen | 33106ES60 |
| Hoechst | 1:1000 | Beyotime | C1027 |

**Table S3 The primer sequences in q-PCR**

| Target | Forword primer | Reverse primer |
| --- | --- | --- |
| GAPDH | ATGGGGAAGGTGAAGGTCG | TAAAAGCAGCCCTGGTGACC |
| Oct4 | GGTTCTCGATACTGGTTCGC | GTGGAGGAAGCTGACAACAA |
| Foxa2 | TACGTGTTCATGCCGTTCAT | CGACTGGAGCAGCTACTATGC |
| Sox17 | TCTGCCTCCTCCACGAA | CAGAATCCAGACCTGCACAA |
| Nkx2.1 | CGGCATGAACATGAGCGGCAT | GCCGACAGGTACTTCTGTTGCTTG |
| Sox2 | GGGGGAATGGACCTTGTATAG | GCAAAGCTCCTACCGTACCA |
| Sox9 | CAGACGCACATCTCCCCCAA | CCCTCTCGCTTCAGGTCAGC |
| Hopx | CCGTCTTCTCTCAGCCACAT | CCAAGCAAGCTTCTTCACCA |
| Sftpb | CTTCCAGAACCAGACTGACTCA | GCTCGGAGAGATCCTGTGTG |
| Sftpc | AGCAAAGAGGTCCTGATGGA | CGATAAGAAGGCGTTTCAGG |

**Table S4 Mass Ratios of Various Components to PM2.5**

| Component Category | Component | Water-extractable fraction | DMSO-extractable fraction |
| --- | --- | --- | --- |
| PAHs（ng/mg） | FLT | 0.0922 | 1.2097 |
|  | PYR | 0.0564 | 1.1385 |
|  | CHR | 0 | 2.6850 |
|  | BaA | 0 | 1.6603 |
|  | BbF | 0.5635 | 10.1423 |
|  | BkF | 0.1178 | 3.7571 |
|  | BaP | 0.3227 | 7.1490 |
|  | B[g,h,i]P | 0.8914 | 8.3065 |
|  | IPY | 1.5984 | 16.8738 |
| Metals（μg/mg） | Hg | 0.1055 | 0.3956 |
|  | Be | 0.0347 | 0.03778 |
|  | As | 23.1660 | 36.4444 |
|  | Se | 11.8533 | 16.8667 |
|  | Al | 370.6564 | 344.4444 |
|  | Cr | 23.9382 | 12.6222 |
|  | Mn | 140.2831 | 123.3333 |
|  | Ni | 10.8237 | 13.8222 |
|  | Cd | 4.4659 | 8.2889 |
|  | Sb | 10.0386 | 16.5778 |
|  | Tl | 1.0592 | 1.3289 |
|  | Pb | 44.5302 | 46.4444 |
| Inorganic Ions (mg/L) | NH4^+^ | 10.1543 | 10.8868 |
|  | F^-^ | 0.1918 | 0.0984 |
|  | Cl^-^ | 5.7222 | 3.1667 |
|  | NO3^-^ | 20.1567 | 21.6692 |
|  | SO4^-^ | 10.5891 | 1.5572 |

**REFERENCES**

Rizzino, A. and Wuebben, E. L., 2016. Sox2/Oct4: A delicately balanced partnership in pluripotent stem cells and embryogenesis. Biochimica et Biophysica Acta (BBA) - Gene Regulatory Mechanisms. 1859(6): 780-791.
